# Efficient targeted multiallelic mutagenesis in tetraploid potato (*Solanum tuberosum*) by transient CRISPR-Cas9 expression in protoplasts

**DOI:** 10.1007/s00299-016-2062-3

**Published:** 2016-10-03

**Authors:** Mariette Andersson, Helle Turesson, Alessandro Nicolia, Ann-Sofie Fält, Mathias Samuelsson, Per Hofvander

**Affiliations:** 1 Department of Plant Breeding, Swedish University of Agricultural Sciences, P.O. Box 101, SE-23053 Alnarp, Sweden; 2 ENEA Research Centre Casaccia, SSPT-BIOAG-BIOTEC, Via Anguillarese, 301, 00123 Rome, Italy; 3 Lyckeby Starch AB, Degebergavägen 60-20, SE-29191 Kristianstad, Sweden

**Keywords:** Genome editing, New plant breeding technique, GBSS, Starch, Amylopectin

## Abstract

**Key message:**

**Altered starch quality with full knockout of** ***GBSS*** **gene function in potato was achieved using CRISPR-Cas9 technology, through transient transfection and regeneration from isolated protoplasts.**

**Abstract:**

Site-directed mutagenesis (SDM) has shown great progress in introducing precisely targeted mutations. Engineered CRISPR-Cas9 has received increased focus compared to other SDM techniques, since the method is easily adapted to different targets. Here, we demonstrate that transient application of CRISPR-Cas9-mediated genome editing in protoplasts of tetraploid potato (*Solanum tuberosum*) yielded mutations in all four alleles in a single transfection, in up to 2 % of regenerated lines. Three different regions of the gene encoding granule-bound starch synthase (*GBSS*) were targeted under different experimental setups, resulting in mutations in at least one allele in 2–12 % of regenerated shoots, with multiple alleles mutated in up to 67 % of confirmed mutated lines. Most mutations resulted in small indels of 1–10 bp, but also vector DNA inserts of 34–236 bp were found in 10 % of analysed lines. No mutations were found in an allele diverging one bp from a used guide sequence, verifying similar results found in other plants that high homology between guide sequence and target region near the protospacer adjacent motif (PAM) site is essential. To meet the challenge of screening large numbers of lines, a PCR-based high-resolution fragment analysis method (HRFA) was used, enabling identification of multiple mutated alleles with a resolution limit of 1 bp. Full knockout of GBSS enzyme activity was confirmed in four-allele mutated lines by phenotypic studies of starch. One remaining wild-type (WT) allele was shown sufficient to maintain enough GBSS enzyme activity to produce significant amounts of amylose.

**Electronic supplementary material:**

The online version of this article (doi:10.1007/s00299-016-2062-3) contains supplementary material, which is available to authorized users.

## Introduction

Potato is ranked as the third most important food crop in the world (Barrell et al. 2013). It has a high nutritional value and yields a high-energy output per hectare. Potato is not only of importance as a food crop, it is also one of the major crops grown for starch production (Ellis et al. 1998). Development of new potato cultivars using traditional cross-breeding is complicated and slow due to tetrasomic inheritance and high heterozygosity of cultivated varieties (Muthoni et al. 2015). Therefore, breeding technologies where only one or a few traits can be introduced into an elite background is of major interest for potato. Genetic modification (GM), by stable integration of genetic material, has been a widely used method in potato research and breeding for a long time (Barrell et al. 2013). However, a long and expensive deregulating process in Europe and elsewhere has restricted commercialisation of the developed GM plants. New breeding techniques where no recombinant DNA is introduced or maintained in the plant chromosomes has shown promise and there is a discussion on whether these techniques result in events that should be regulated as GMO (Jones 2015; Waltz 2016).

Starch produced from potatoes has many uses, both in food and technical applications, and is often chemically or physically modified to certain specifications (Ellis et al. 1998). Some of the processes for chemically or physically modified starches cause environmental concerns and it would be advantageous for these processes to be replaced by starch modified *in planta*. Starch is a mixture of two components, amylose and amylopectin. Changing the ratio of these two components greatly alters the properties of the starch. Many applications would gain advantages by having a change in proportion or a starch containing only one of the two components (Zeeman et al. 2010). The so-called waxy genotype producing essentially only amylopectin starch was first identified in maize and the corresponding locus was identified (Klosgen et al. 1986; Shure et al. 1983). One single enzyme was found responsible for the synthesis of amylose, granule-bound starch synthase (GBSS). In potato, as in most other plants, the GBSS enzyme is encoded by a single locus (*GBSSI*) having four alleles in the cultivated potato. High amylopectin potatoes (Waxy potato) have been developed by silencing of the *GBSSI* gene function through the use of antisense technology (Kuipers et al. 1994; Visser et al. 1991), RNAi technique (Andersson et al. 2003) or traditional mutational breeding (Muth et al. 2008). Today, only the Waxy potato variety Eliane™, developed by radiation-induced mutations (Hovenkamp-Hermelink et al. 1987; Muth et al. 2008), is grown commercially.

Gene silencing is widely used in plant research and breeding, to study gene functions as well as to develop new cultivars. Recently, site-directed mutagenesis (SDM) has been applied for this purpose. Compared to chemical and physical mutagenesis, which is random with multiple mutations introduced throughout the genome, the SDM techniques are designed to be target specific (Quetier 2016). The constructs for SDM, can be stably inserted into the genome by transformation, or be transiently expressed to introduce in vivo mutations. Zinc finger nuclease technology (ZFN), TAL effector nucleases (TALEN) and clustered regularly interspaced short palindromic repeat (CRISPR) and CRISPR-associated protein 9 (Cas9) are the main SDM techniques currently in use (Schiml and Puchta 2016). Development of CRISPR-Cas9 has received much attention lately due to being a more user-friendly and cost-efficient technique of producing target-specific constructs compared to ZFN and TALEN. The CRISPR-Cas9 technique was first reported as successful in higher plants in 2013 (Li et al. 2013; Nekrasov et al. 2013; Shan et al. 2013). The method is based on a short single-guide RNA (sgRNA), with a 20 bp guide sequence complementary to a target region, a promoter and a sgRNA scaffold, which in combination with a Cas9 nuclease (Jinek et al. 2012) can induce mutations in a target region of choice. The resulting double strand break (DSB) is repaired by the cell’s own repair mechanism, either through non-homologous end joining (NHEJ) or homologous recombination (HR) (Britt 1999). NHEJ is error prone and often leads to random-sized inserts or deletions (indels), which may cause a knockout of gene function.

Recently, the first studies using TALEN and CRISPR-Cas9 to induce mutations in potato were published (Butler et al. 2015, 2016; Clasen et al. 2016; Nicolia et al. 2015; Sawai et al. 2014; Wang et al. 2015). In a TALEN study, stable transformation was used for studying the disruption of a sterol side chain reductase 2 (StSSR2). In that study, two lines with confirmed indels were described in detail of which one line was concluded to have all four alleles mutated (Sawai et al. 2014). Further, a transient method for TALEN expression was developed and induced mutations were verified in the target gene, ACETOLACTATE SYNTHASE (ALS), in 2 out of 20 regenerated shoots (Nicolia et al. 2015). In that study, no shoots with multiple mutated alleles were described. Shortly after, another study used transiently expressed TALEN to knockout a VACUOLAR INVERTASE gene (*Vlnv*), at a mutation frequency between 2 and 16 % and with two-thirds of the regenerated plants having multiple alleles mutated (Clasen et al. 2016). CRISPR-Cas9 has, in two different studies, been shown to induce mutations in potato by *Agrobacterium*-mediated stable transformation. In the first study, a gene encoding an Aux/IAA protein, *StIAA2*, was targeted in a double haploid potato cultivar (Wang et al. 2015) while in the second study with this method, the ALS gene was targeted in both a diploid and tetraploid potato (Butler et al. 2015). With confirmed stable integration, the studies yielded mutation rates of 83 and 60 % of regenerated lines, respectively. Furthermore, lines with mutations in multiple alleles were detected in both cases. Most recently, TALEN and CRISPR-Cas9 were stably introduced targeting ALS and using a geminivirus-mediated guide, to facilitate designed mutations (Butler et al. 2016). 

In this work, we have developed full gene knockouts of tetraploid potato using transient expression of the CRISPR-Cas9 system designed to target a *GBSS* gene. This is substantiated by genetic analysis of the target gene and phenotypical assessment of starch quality. Using transient expression of targeted CRISPR-Cas9 in tetraploid potato, mutations in all four alleles were obtained without stable integration of DNA and the laborious subsequent crossing for re-establishment of a potato genotype with desired agronomical properties.

## Results and discussion

### Design of CRISPR-Cas9 constructs

**Fig. 1:**
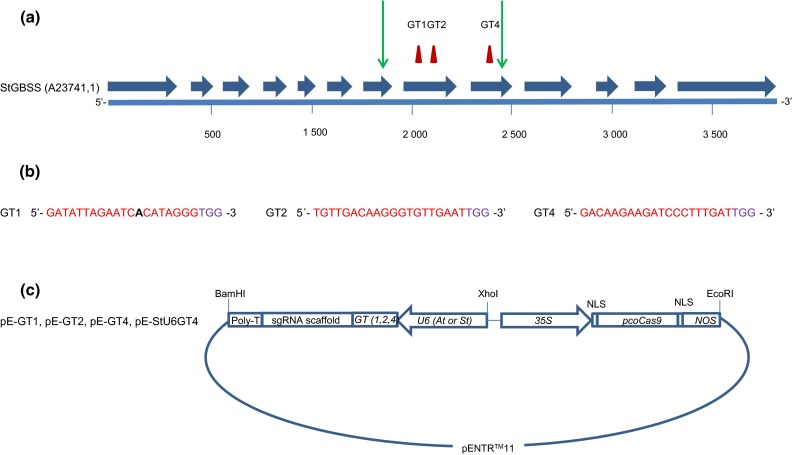
Design of CRISPR-Cas9 constructs targeting the *GBSS* gene. **a** Illustration of *Solanum tuberosum GBSS* gene structure (GenBank accession no. A23741.1). Exons 1–13 are marked with *blue arrows*, outer boundaries of fragments amplified for sequence determination of allelic variation are marked with *green arrows* and CRISPR-Cas9 target regions GT1, GT2 and GT4 are marked with *red triangles*. **b** Target regions GT1, GT2 and GT4 are in red and PAM-site in purple. Allelic variation in GT1 marked in *black bold text*. **c** Constructs designed for CRISPR-Cas9-mediated induction of mutations in *StGBSS*; from left to right: terminator (poly-T), sgRNA scaffold, guide sequence (GT1, GT2 or GT4), *U6* promoter of either *Arabidopsis thaliana* or *Solanum tuberosum* origin, 35S promoter of cauliflower mosaic virus origin (CaMV), nuclear localization sequence (NLS), plant codon-optimized Cas9 gene, NLS and nopaline synthase terminator (NOS) in vector pENTR™11. The elements shown in the illustration are not to scale in relation to each other

To determine allelic variation in parts of the *GBSS* gene in the tetraploid potato variety Kuras, fragments covering exon 8 and parts of exon 9, as well as adjacent introns (Fig. 1a) were amplified and sequenced. The different alleles were found to be highly similar to each other as well as to a published *GBSS* gene (Supplementary Fig. S1). Out of the four alleles, only one of them had a base pair (bp) variation in exon 8 with an adenine (A) → guanine (G) shift and two alleles had an adenine (A) → guanine (G) shift at one position in exon 9 (Supplementary Fig. S1). Two target regions in *GBSS* exon 8 were selected and named GT1 and GT2 and one target region in exon 9 was selected and named GT4. GT1 spanned the region in exon 8 having the allelic variation (Fig. 1a, b). Two different promoters were chosen for driving the sgRNA expression of the corresponding guide sequences. *U6* promoters have been the most commonly used type of promoter for driving expression of sgRNA in plants (Bortesi and Fischer 2015). Although *Arabidopsis thaliana* has been the preferred origin of *U6* promoter, in some studies an endogenous *U6* promoter has been tested with a positive influence on the frequency of induced mutations (Sun et al. 2015). The Arabidopsis *U6* promoter was used in combination with all three guide sequences, respectively. To study the effect of an endogenous promoter, a *U6* promoter of *Solanum tuberosum* origin (StU6) (Guerineau and Waugh 1993) was combined with the GT4 guide sequence. The use of an *StU6* promoter driving sgRNA in potato was also reported in the CRISPR-Cas9 study by Wang et al. 2015, although this promoter originated from a different StU6 gene (Wang et al. 2015). *Solanum tuberosum U6* small nuclear RNAs are members of a large gene family with promoter sequence variation (Guerineau and Waugh 1993). Naked vector DNA (pE-GT1, pE-GT2, pE-GT4 and pE-StU6GT4) with respective guide sequence GT1, GT2 and GT4 and plant codon-optimized Cas9 (pcoCas9) (Li et al. 2013) (Fig. 1c) was purified for protoplast transfection. To evaluate if Cas9 and sgRNA expressed from separate vectors had an influence on mutation efficiency, purified vector DNA containing pcoCas9 and sg-GT1 cassettes were co-expressed in the study.

### Protoplast transfection and regeneration

DNA was transiently expressed, with the aim of inducing mutations in the *GBSS* gene. Transient expression was chosen to facilitate the generation of genotypes with induced mutations without stable integration of DNA in the genome. Potato could easily be transformed using *Agrobacterium tumefaciens* generating a high number of transgenic shoots in a short time period (Andersson et al. 2003; Visser et al. 1989). However, the subsequent outcrossing of inserted T-DNA would disrupt the genetic context of the modified potato genotype due to its heterozygosity and tetrasomic inheritance.

Expression of the four sgRNA-pcoCas9 constructs, pE-GT1, pE-GT2, pE-GT4 and pE-StU6GT4, as well as the combined expression of GT1-sgRNA and pcoCas9 from separate vectors was achieved through PEG-mediated protoplast transfection. The protoplast isolation and transfection method used was essentially as described by Nicolia et al. 2015, where a method to induce mutations in potato via TALEN was developed (Nicolia et al. 2015). In this study, the protocol was further developed and adapted for CRISPR-Cas9. Besides the four different constructs used, transfection parameters were varied, such as amount of DNA, incubation time, concentration of protoplasts and concentration of PEG (Table 1). After transfection, the protoplasts were embedded in alginate until callus was formed. Approximately, 4 weeks after transfection, the calluses were released and incubated in liquid media for an additional 2 to 4 weeks to allow further development. The enlarged calluses were then transferred to solid medium for shoot development.

**Table 1:** Protoplast transfection experimental setup and results thereof

Guide sequence	Promoter	PEG (%)	Transfection time (min)	DNA (µg)	Protoplasts (/mL)	Number regenerated mutated lines (number analysed)	Frequency mutated lines (%)
GT1	*A.t.U6*	12.5	3	10	80 000	8 (161)	5.0
GT1	*A.t.U6*	25	3	5	80 000	7 (85)	8.2
GT1 + Cas9^a^	*A.t.U6*	25	3	5 + 5	80 000	4 (130)	3.1
GT1	*A.t.U6*	40	30	15	50 000	5 (43)	11.6
GT2	*A.t.U6*	12.5	3	10	80 000	12 (286)	4.2
GT2	*A.tU6*	25	3	5	80 000	5 (108)	4.6
GT2	*A.t.U6*	40	30	15	50 000	2 (79)	2.5
GT4	*S.t.U6*	12.5	3	10	80 000	18 (188)	9.6
GT4	*A.tU6*	25	3	5	80 000	21 (407)	5.2
GT4	*S.tU6*	25	3	5	80 000	44 (426)	10.3
GT4	*S.t.U6*	40	30	15	50 000	3 (138)	2.2

Mutation frequency (%) calculated for each experiment based on mutated lines detected using high-resolution fragment analysis by the total number of regenerated lines analysed

^a^GT1 and Cas9 expressed from separate vectors

Shoot regeneration was similar in the 12.5 and 25 % PEG experimental setups, where a first shoot emerged 11–15 weeks after transfection (Supplementary Fig. S2). After 6 months, 34–43 % of all developed calluses from the pE-GT2 and pE-GT4 expressions at the 25 % PEG experimental setup had produced a shoot. Shoot development was peaking between months 4 and 7, but shoots continuously emerged up to one year after transfection (Supplementary Fig. S2). In two out of three experiments with the highest PEG concentration of 40 % and with the lower density of 50,000 protoplasts/mL culture medium, the shoot regeneration was delayed and depressed (Supplementary Fig. S2). High density of protoplasts or use of feeder cells is generally believed to stimulate cell division, by releasing stimulating substances into the medium (Xu and Xue 1999). However, this needs to be balanced since a high density of viable protoplasts will hamper distinct separation of individually developing calluses.

While the majority of the viable shoots developed into plantlets undistinguishable from the parental phenotype, some of the regenerated shoots failed in elongation and hence development into plantlets. Based on all regenerated shoots in this study, an average of 25 % were stunted in growth and eventually died. In previous studies where silencing of *GBSS* in potato has been the objective, no impact on plant growth has been reported (Hofvander 2004; Kuipers et al. 1994). Therefore, it is not likely that the cause for a stunted growth is a complete knockout of the GBSS enzyme function. Plants regenerated from potato leaf protoplasts can lead to somaclonal variation, which might be a plausible explanation for the observed negative effect on plant development in this study (Larkin and Scowcroft 1981; Shepard et al. 1980). Although a substantial share of shoots was discarded due to failed development, the regeneration rate of the method ensures a vast number of shoots to select from and where the subsequent selection process does not differ from other plant breeding methods.

### High-resolution fragment analysis (HRFA) for screening of mutations

**Fig. 2:**
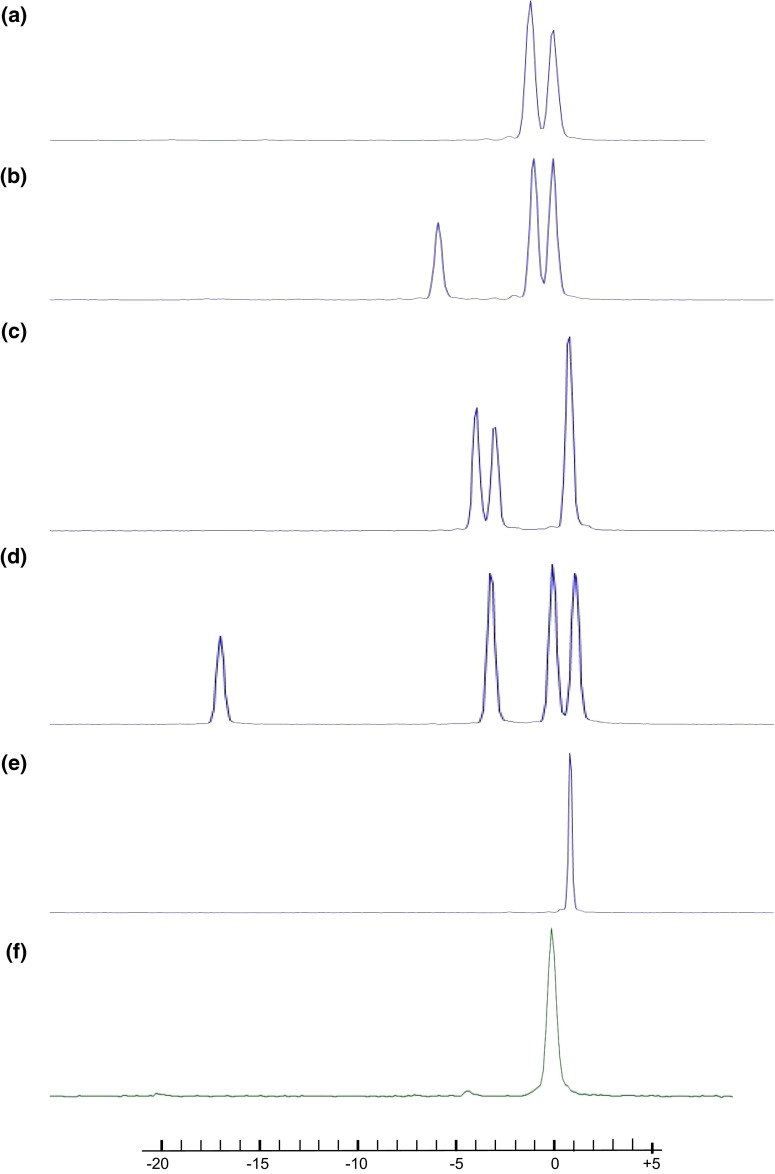
Detection of induced mutations in the *GBSS* gene using high-resolution fragment analysis (HRFA). Elution point of wild-type fragment is set to 0 on the bp scale. **a** Line P1003 having 1–2 alleles mutated in the GT1 target region. **b** Line P1004 having 2–3 alleles mutated in the GT1 target region. **c** Line P7021 having 4 alleles mutated in the GT2 target region. **d** Line P11088 having 3 alleles mutated in the GT4 target region. **e** Line P24023 having 4 alleles mutated in the GT4 target region. **f** Wild type

Efficient screening methods for induced mutations are crucial to analyse a large number of genome-edited regenerated plants. Here, we used a high-resolution fragment analysis, HRFA, with 96-format DNA extraction, PCR amplification and capillary electrophoresis. A leaf sample of each regenerated shoot was subjected to DNA isolation followed by PCR amplification covering the respective target sites using a fluorescently labelled forward primer. Capillary electrophoresis was applied to the amplified reactions for separation with high resolution and to fluorescently detect PCR products differing in size. The method was highly sensitive and indels as small as 1 bp were detected (Fig. 2a). In addition, multiple mutated alleles, with indels of differing size, were distinguished (Fig. 2b–d). It was not possible to detect mutations yielding a nucleotide substitution with the method. However, since the majority of the nucleotide exchanges only result in an amino acid substitution in the corresponding protein, these mutations were of less interest since a complete knockout of enzyme activity was intended. A limitation of the method was that alleles having same sized indels did co-elute and could not be resolved. A corresponding wild-type fragment labelled with a different fluorescent dye was included in the analysis (Fig. 2f). Loss of wild-type size fragment signal in the regenerated lines was accordingly a confirmation that all four alleles had received mutations. The applied HRFA method can easily be adapted to multiplex analysis of samples with more than one gene targeted by designing PCR amplicons differing in size and labelled with different fluorophores.

A range of different techniques are in use for screening plants for induced mutations. Detection of indels using PCR and capillary electrophoresis is used here and elsewhere (Ramlee et al. 2015; Yang et al. 2015); in comparison to the more generally used DNA sequencing method (Bortesi and Fischer 2015) it has a much shorter time span from sampling to identification of mutants. Furthermore, screening polyploid plants by sequencing, and verifying coverage of all alleles might lead to analysis of many replicates. A 454 sequencing approach or similar might give a more reliable result, but is still costly in comparison to HRFA. Other commonly used methods for detection of mutation are high-resolution melt analysis (HRM) (Wittwer et al. 2003) as well as cleaved amplified polymorphic sequences (CAPS) (Konieczny and Ausubel 1993), a method based on the loss of function of a restriction site located in the predicted cleave site in the target region. CAPS are efficient and easy to use, but limit the target guide design and the number of target sites that can be identified in a gene. The use of a heteroduplex mobility assay is yet another alternative, but the allelic specificity and sensitivity of the method is lower, having a published resolution limit of 3 bp indels (Delwart et al. 1993; Ito et al. 2015).

### Identification of mutated lines

To validate CRISPR-Cas9 functionality when transiently expressed in protoplasts of a tetraploid potato cultivar and to estimate the mutation efficiency of eleven different experimental setups, a total of 2051 regenerated shoots were analysed by HRFA. As shown in Table 1, directed mutations were induced to a frequency of 2.2–11.6 %. No striking difference could be found between the three *GBSS* sites targeted, GT1, GT2 and GT4, yielding mutation frequencies of 8.2, 4.6 and 5.2 %, respectively, in the 25 % PEG experimental setup. Hence, the CRISPR-Cas9 technique was not very sensitive to target region and corresponding guide sequence chosen in this study. Similar findings have previously been published; for example, in a study where CRISPR-Cas9 was transiently expressed for targeted genome editing in rice (Xie and Yang 2013).

Even though the mutation frequency using pE-GT1 increased with a higher PEG concentration, DNA amount and incubation time used (5.0, 8.2 and 11.6 % at 12.5, 25 and 40 % PEG experimental setups, respectively, Table 1), the tendency was not consistent among the different guide sequences studied. pE-GT2 expressed at 12.5, 25 and 40 % PEG experimental conditions, resulted in mutation frequencies of 4.2, 4.6 and 2.5 %, respectively. In the experiment where Cas9 and the GT1-sgRNA were expressed from separate vectors (DNA ratio 1:1 (w/w)), a frequency of 3.1 % was found which was somewhat lower (2.6-fold) than when expressed from a single construct at the same transfection conditions (Table 1).

Previous studies have shown that the choice of promoter driving the guide sequence could have an impact on the mutation frequency (Sun et al. 2015). It has also been shown that the strength of the promoter can have an effect on target specificity, and a high concentration of sgRNA-Cas9 transcripts can increase off-target mutations (Hsu et al. 2013; Pattanayak et al. 2013). The *U6* promoters used in this study yielded only a small difference on the mutation frequency. GT4 guide sequence driven by either the Arabidopsis or potato *U6* promoter yielded a mutation frequency of 5.2 and 10.3 %, respectively in the 25 % PEG experimental setup.

In general, the mutation frequencies found in this study were in the same range as has previously been observed using in vivo-directed mutagenesis in potato. In the two TALEN-studies published, where transient expression was used for directed mutagenesis in potato, the frequencies were 10 % when 25 % PEG was used for transfection (Nicolia et al. 2015) and 2–16 % using 40 % PEG for transfection (Clasen et al. 2016). Not surprisingly, a higher mutation frequency was found in the studies with stable integration of CRISPR-Cas9, resulting in 3–60 % (Butler et al. 2015) and 83 % (Wang et al. 2015), respectively. Even though stable integration of CRISPR-Cas9 results in a higher mutagenesis rate, a transient expression of CRISPR-Cas9 would be preferable for new potato genotype development, particularly when taking into account the efficient application of transient expression in the generation of mutations as shown in this study.

### Mutation validation and characterization

**Fig. 3:**
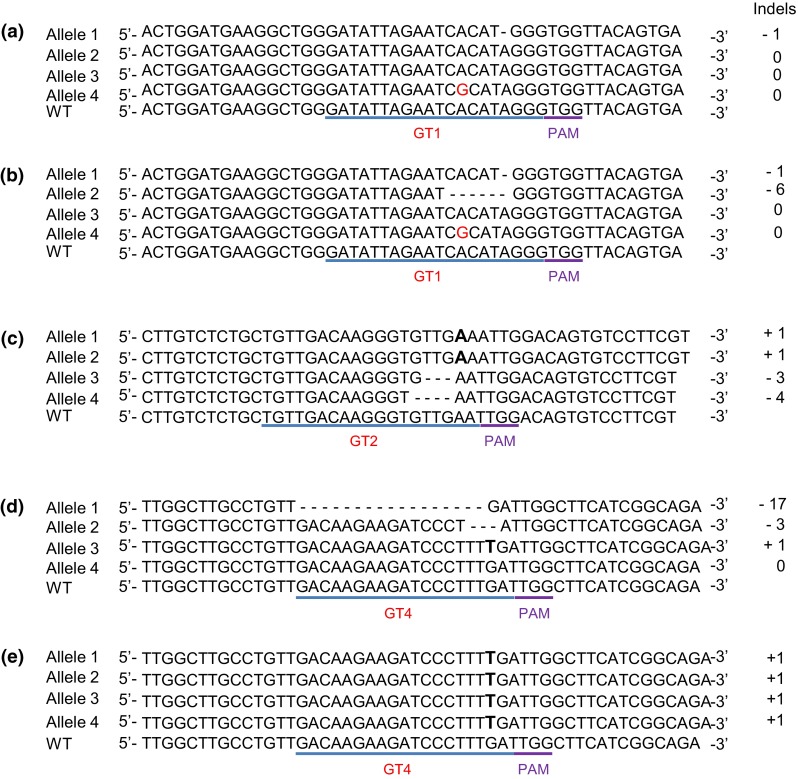
Genotyping of lines with induced mutations. Shown are mutations in individual alleles aligned to a corresponding wild-type (WT) fragment, determined by DNA sequencing. Deleted nucleotides are shown with hyphens and inserted nucleotides are shown in *bold*. **a** P1003 having at least 1 allele and maximum 2 alleles mutated in the GT1 target region. **b** P1004 having 2 alleles mutated in the GT1 target region. **c** P7021 having 4 alleles mutated in the GT2 target region. **d** P11088 having 3 alleles mutated in the GT4 target region. **e** P24023 having 4 alleles mutated in the GT4 target region

Out of 129 lines, identified as mutated by the HRFA analysis, 29 were subjected to genotyping to determine the exact size and location of the mutations (Fig. 3 and supplementary Fig. S3). PCR amplifications covering the respective target region in the individual lines were cloned and analysed by DNA sequencing. The genotyping corresponded well to the size of the indels and number of alleles mutated found with the HRFA analysis (Figs. 2, 3). Most deletions were starting at position 4 adjacent to the PAM site (Fig. 3 and supplementary Fig. S3), but deletions starting at position 2, 3 or 5 (Supplementary Fig. S3, line P8006, P7021, P11088, P2058 and P24100) as well as after the PAM site in position 4 (P10084) were also detected in the mutated lines studied. The majority of the mutations consisted of small deletions of 1–10 bp. Six of the sequenced mutated lines were confirmed to have a single-bp insertion (Supplementary Fig. S3) and additionally three lines had larger inserts of 34, 125 and 236 bp, respectively, which were found to be integration of random parts of vector DNA at the predicted target site (data not shown). Small indels of 1 bp seem to be the most common mutations caused by DSB repair from CRISPR-Cas9-targeted mutagenesis (Feng et al. 2014). In addition, mutations where a few bp towards the PAM site have been deleted are generally observed both in potato and in other plant species (Butler et al. 2015; Ito et al. 2015; Li et al. 2013; Wang et al. 2015).

Integration of vector DNA in the target site during CRISPR-Cas9 transient expression is not frequently published. However, in the study by Clasen et al. 2016, 60 % of the regenerated shoots were found to have vector DNA inserted when TALEN-modified plants were studied using PCR analysis, but the exact nature of vector DNA insertion was not described. The 10 % frequency of vector DNA inserts in our study was found integrated in the predicted Cas9 cut site in lines expressing pE-GT2 or pE-GT4. No obvious homology between the vector DNA and the cleavage site could be found, which would indicate a random integration of available DNA upon repair of the double strand break by NHEJ. It is desirable to avoid integration of vector DNA as this would be considered as non-intended integration of foreign DNA from the perspective of how the produced genotypes can be further handled and studied. At a low rate of integration, plants containing vector DNA could simply be discarded. However, a higher rate of integration would warrant a further development of the method with the aim to decrease number of lines with vector integration.

The GT1 guide sequence, which was designed as spanning the A → G allelic variation, was not found to induce mutations in the G-allele (Fig. 3a–b, Supplementary Fig. S3). CRISPR-Cas9 has been shown to tolerate certain mismatches. However, guide sequences with mismatches closer to the PAM region are generally not functional (Hsu et al. 2013; Xie and Yang 2013). The mismatch in the G-allele in our study was at position 7 in relation to the PAM sequence, which due to its location quite close to the PAM site confirms previously published results on a low tolerance of mismatches in the region close to the PAM sequence.

**Fig. 4:**
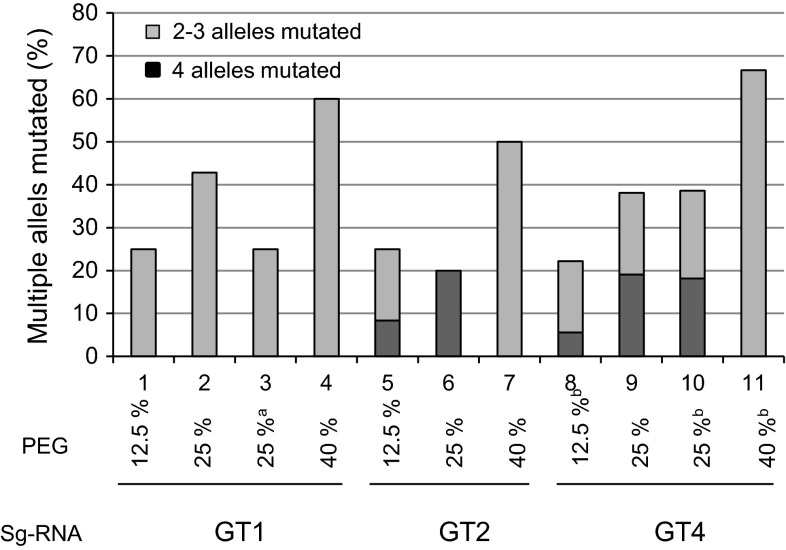
Frequency of mutated lines with multiple alleles targeted. The result is calculated based on all mutated lines regenerated in each experiment (%) analysed with high-resolution fragment analysis. ^a^GT1 and Cas9 expressed from separate vectors. ^b^ *Solanum tuberosum* U6 promoter driving the sgRNA expression. *n* = 2–44

The described method in this study was found to induce mutations in more than one allele with a high frequency. Based on the different experimental setups, between 20 and 67 % of the mutated lines contained multiple mutated alleles. The rate might be higher though, since mutations of equal size in up to three alleles were difficult to discern. This study indicated that the frequency of multi-allelic mutagenesis could be dependent on varying experimental setups of PEG concentration (Fig. 4). In 15 of the lines, HRFA showed that all 4 alleles were mutated. Six of those lines were subjected to sequencing, where no wild-type *GBSS* fragment was found, confirming the HRFA results. In the most successful experiment, with GT4 driven by the potato promoter in the 25 % PEG experimental setup, approximately 2 % of regenerated lines had mutations in all four alleles (Table 1; Fig. 4) showing the capacity of this method to fully knockout a gene in a polyploid plant such as potato in a single transfection.

### Starch phenotyping

**Fig. 5:**
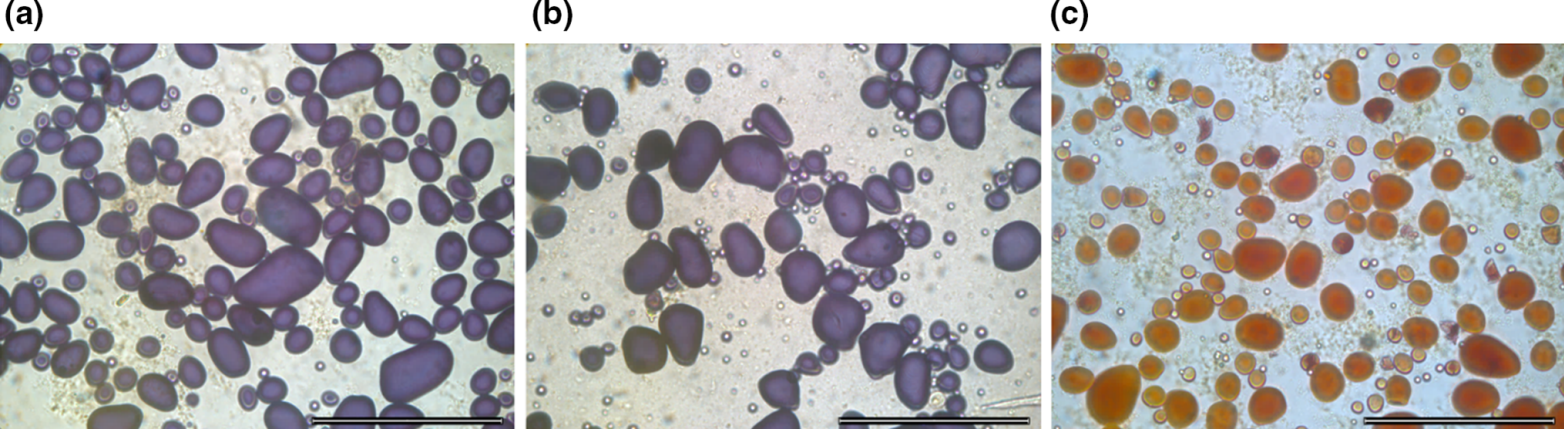
Starch characterization. Starch from microtubers stained with iodine and visualized under light microscopy. Starch stained *blue* contains amylose and starch stained *red*-*brown* lacks amylose. **a** Wild type. **b** Line P11088 having 3 alleles mutated. **c** Line P24023 having 4 alleles mutated. *Scale bar* 100 µm

Line P11088 with three *GBSS* alleles mutated and line P24023 with four alleles mutated were subjected to starch phenotyping. In vitro microtubers were produced from which starch was studied under light microscopy. Reduction of GBSS enzyme activity leads to a starch with altered amylose synthesis and a concomitant increase in the amylopectin/amylose ratio. An amylopectin starch can be identified using iodine staining, resulting in starch granules that are red-brown in colour, compared to starch containing amylose which stains blue. In this study, we found that starch from a three-allele mutated line stained blue using iodine, hence a significant amount of amylose is produced even though only one *GBSS* allele is functional. This shows that GBSS enzyme activity is available in large excess in potato, which is in line with results from previous publications (Visser et al. 1991). In the line with all four alleles mutated, the starch was confirmed as being of amylopectin quality by the red-brown staining with iodine (Fig. 5), demonstrating the knockout of GBSS enzyme activity.

## Conclusion

This study presents the successful application of CRISPR-Cas9 to fully knockout a gene function in a tetraploid plant in one round of transfection, without stable introduction of DNA into the genome. Targeted mutations via in vivo methods are of significant importance for future potato breeding since one or a few traits can be added in a commercially interesting potato variety. This can then be done without disrupting the valuable overall heterozygous genetic context by avoiding additional sexual crosses. In this study, we targeted three different regions of the same gene, using two different promoters for driving the guide sequences as well as three different transfection conditions. The different experiments all yielded high mutation frequencies, in the same order of magnitude, demonstrating the robustness of the CRISPR-Cas9 technique for potato research and breeding. Here, we also demonstrated the use of this new breeding technology to develop a trait of commercial interest, an amylopectin potato starch, with uses in both food and technical applications.

## Materials and methods

### Allelic variation of target region

Genomic DNA from leaf tissue of Kuras was extracted using Gene Jet Plant Genomic DNA Purification Mini Kit (Thermo Fisher Scientific, Waltham USA), and used to amplify two fragments of 508 and 684 bp of the *GBSS* gene (Supplementary Fig. S1). 250 ng of DNA was used as template in a PCR with 0.5 µmol of primers (Supplementary Table S1) StGBSSExf and StGBSSExr, and primers StGBSSExon3f and StGBSSExon3r (Sigma-Aldrich, St. Louis, USA), Phusion HF polymerase, dNTP, and HF buffer (Thermo Fisher Scientific, Waltham, USA) according to the supplier’s instructions. The PCR was as follows: 98 °C for 1 min, 30 cycles at 98 °C for 10 s, 64 °C for 10 s, 72 °C for 15 s and final extension at 72 °C for 10 min.

The PCR product was ligated to pJET1.2/blunt using a CloneJET PCR Cloning Kit (Thermo Fisher Scientific, Waltham, USA) following transformation to One Shot^®^ TOP10 Chemically Competent *Escherichia coli* (Invitrogen, Carlsbad, USA). After overnight incubation at 37 °C on LB plates containing 100 μg/mL ampicillin, plasmids were purified from 12 randomly picked colonies and insert sequenced (GATC Biotech, Konstanz, Germany).

### Vector construction

Target regions in GBSS exon 8 and 9 were identified manually or using a publically available CRISPR-design web-based tool (http://crispr.mit.edu/) with mismatches mapped against the *A. thaliana* genome. Three target regions were selected and corresponding guide sequences were named GT1, GT2 and GT4 (Fig. 1a, c). Each target region of 20 bp was located directly upstream of a 5′-NGG-3′ PAM site and with, except for GT2, the nucleotide G in the 5′ end. GT1, 5′-GATATTAGAATCACATAGGG-3′, and GT2, 5′-TGTTGACAAGGGTGTTGAAT-3′, are located within exon 8, with GT1 spanning a region with an allelic variation, and GT4, 5′-GACAAGAAGATCCCTTTGAT-3′, is located in exon 9 (GenBank accession no. A23741.1). The corresponding guide sequences were synthetically produced (Eurofins Genomics, Ebersberg, Germany) together with a 5′ XhoI site, a *U6* promoter of *Arabidopsis thaliana* (GenBank accession no. X52527.1) or *Solanum tuberosum* origin (GenBank accession no. Z17290.1) and SgRNA described elsewhere (Li et al. 2013) and received cloned in standard vector pEX-A2. For GT2, an additional G nucleotide was added directly upstream of the guide sequence. The synthesized fragments were cloned together with a plant codon-optimized Cas9 gene (pcoCas9) driven by a 35S promoter (kindly provided by Professor Jen Sheen, Harvard Medical School, Boston, USA) (Fig. 1d). The guide sequence–SgRNA complex was digested with XhoI and BamHI (located in vector pEX-A2) and Cas9 (Li et al. 2013) was digested from pHBT35sPPDKCas9 with XhoI and EcoRI. The two fragments were purified using Gene Jet Gel extraction kit (Thermo Scientific, Waltham USA) and ligated to a BamHI- and EcoRI-digested standard Gateway^®^ entry vector pENTR11 (Invitrogen, Carlsbad, USA) in a three-fragment cloning. All constructs were transformed to One Shot^®^ TOP10 Chemically Competent *E. coli* (Invitrogen, Carlsbad, USA) and named pE-GT1, pE-GT2, pE-GT4 and pE-StU6GT4, respectively. Additionally, Cas9 and target guide GT1 expression cassettes in their original vectors were transformed to One Shot^®^ TOP10 Chemically Competent *E. coli* (Invitrogen, Carlsbad, USA) for co-expression. Transfection quality vector DNA was extracted from confirmed bacterial colony with Qiagen Plasmid Mini Kit (Qiagen, Hilden, Germany) according to the manufacturer’s instructions.

### Plant material

Potato cultivar Kuras was propagated in 1X Murashige and Skoog (MS) medium (pH 5.8) including vitamins, 3 % sucrose, 8 µM silver thiosulphate (STS) and 0.7 % phytoagar (Duchefa, Haarlem, The Netherlands) in a controlled environmental chamber at 24 °C for 16 h in light and 20 °C for 8 h in dark.

### Transient expression of isolated protoplasts

Protoplast isolation, transfection and regeneration of shoots were essentially performed as previously described (Nicolia et al. 2015). Protoplasts were isolated from top leaves of 5–6-week-old seedlings. Transient expression of pE-GT1, pE-GT2 and pE-GT4 as well as expression of the GT1 target region complex and pcoCas9 from separate vectors were made through a PEG-mediated transfection method. Transfection was performed at room temperature using 1.0 × 10^5^ or 1.6 × 10^5^ protoplasts in 100 µl of 12.5, 25 or 40 % PEG4000 (Sigma-Aldrich, St. Louis, USA) and 5, 10 or 15 µg of purified DNA and with an incubation time of 3 or 30 min (Table 1). After transfection, the protoplasts were embedded in 2 mL of culture medium–alginate solution (Sigma-Aldrich, St. Louis, USA) and incubated at 25 °C in darkness until first cell division took place. During the following 2 weeks, the light was stepwise increased to reach 10 µmol/m^2^/s (Memmert, Schwarbach, Germany) until calluses visible to naked eye had been formed. Approximately 3 to 4 weeks after transfection, each callus was released and incubated in liquid media (Medium G) for two to four additional weeks resulting in further callus development and shoot induction. The enlarged calluses were then transferred to solid medium (Medium H) for shoot development (Nicolia et al. 2015).

### Primary screening using high-resolution fragment analysis

Leaf tissue samples from 2- to 5-week-old plantlets, approximately 5 mm in diameter, were picked and placed in a 96 DeepWell plate on ice together with a 5-mm steel bead. 500 µl of lysis buffer (100 mM Tris, 50 mM EDTA and 1 % SDS, pH 9.0) was added to each well and the tissue was homogenized in a Retsch Mixer Mill MM400 (Retsch GmbH, Haan, Germany) (30 s, 30 Hz). DNA was extracted from 200 µl of cleared lysate in a QIAcube HT extraction robot using a QIAamp 96 DNA QIAcube HT Kit (Qiagen, Hilden, Germany) utilizing the standard DNA extraction protocol provided by the supplier with addition of RNAseA to a final concentration of 0.1 mg/mL in the elution buffer (AE). From each 96-well plate, DNA quantity and quality of one row (8 samples) was controlled by measuring the concentration by NanoDrop™ and visually inspected on a 1 % agarose gel. PCR was performed on the extracted DNA in a reaction containing 1X Phusion HF buffer, 0.2 mM dNTPs, 0.25 µM of each primer, 0.2 units Phusion polymerase, 0.5 µl of DNA extract and water to a final volume of 10 µl (Thermo Fisher Scientific, Waltham, USA). The forward primers were labelled at the 5′ end with the fluorescent dyes FAM (Sigma-Aldrich, St. Louis, USA) or VIC (Thermo Fisher Scientific, Waltham, USA); StGBSSexon1f-FAM, StGBSSexon1f-VIC, StGBSSexon1r, StGBSS(GT4)f-FAM, StGBSS(GT4)f-VIC and StGBSS(GT4)f (Supplementary Table S1).

The cycling conditions were 98 °C for 1 min, 30 cycles of 98 °C 10 s, 64 °C 10 s, 72 °C 15 s and a final extension of 72 °C for 10 min. A volume of 0.5 µl of the PCR product (diluted 1:20) was mixed with 0.5 µl of control PCR fragment (diluted 1:20) and 9.0 µl of Hi-Di™ Formamide (Thermo Fisher Scientific, Waltham, USA) denatured at 95 °C for 3 min and cooled on ice. Analysis was performed on a 3500 Genetic analyser (Thermo Fisher Scientific, Waltham, USA) according to the manufacturer’s instructions with the 3500 Series Data Collection Software 3. The respective control fragment was amplified from wild-type Kuras with the VIC-labelled forward primer.

### Genotyping of mutated lines

PCR amplifications covering the respective target region in the individual lines were performed using 0.5 µl of isolated DNA, 0.25 µM of each primer and Phusion polymerase (Invitrogen, Carlsbad, USA) according to the manufacturer’s instructions. The primers used were StGBSSexon1f,StGBSSexon1r,3′ and StGBSS(GT4)f, StGBSS(GT4)r (Supplementary Table S1). The PCR conditions were as follows: 98° for 1 min, 30 cycles of 98 °C for 10 s, 64 °C for 10 s, 72 °C for 15 s and a final extension of 72 °C for 10 min. The PCR products were ligated to pJET1.2/blunt using a CloneJET PCR Cloning Kit (Thermo Fisher Scientific, Waltham, USA) following transformation to One Shot^®^ TOP10 Chemically Competent *E. coli* (Invitrogen, Carlsbad, USA). After overnight incubation at 37 °C on LB plates containing 100 μg/mL ampicillin, plasmids were isolated and sequenced from 12 randomly picked colonies of each line (GATC Biotech, Konstanz, Germany).

### Phenotyping of starch

Analysis of amylopectin/amylose ratio using iodine staining of starch from microtubers was done as previously described (Andersson et al. 2003).

#### **Author contribution statement**

MA. and P.H. conceived and designed the research. M.A., P.H., H.T., A.N. and A-S.F. designed and conducted experiments. M.S. supervised the research. M.A., P.H. and H.T. wrote the manuscript. All authors read and approved the manuscript.

## Electronic supplementary material

Below is the link to the electronic supplementary material.

Supplementary Allelic variation *GBSS*. Alignment of the four *GBSS* alleles of Kuras, determined by Sanger sequencing and compared to a publically available *GBSS* sequence (Gen Bank, accession no. A23741.1). (a) exon 8. (b) part of exon 9. Variations are marked with a red box. (PPTX 69 kb)

Number of shoots regenerated per month after transfection. Guide sequences corresponding to target regions GT1, GT2 and GT4 at different transfection conditions. Shown within the parentheses’ is the PEG concentration used in the experiment. A total of 160,000 protoplasts were transfected in the 12.5 and 25 % PEG experimental setups while 100,000 protoplasts were transfected using the 40 % PEG experimental setup. (PPTX 167 kb)

Genotyping of individual alleles. Lines with induced mutations in GT1, GT2 and GT4 target regions are presented. Deleted nucleotides are shown with hyphens and inserted nucleotides are shown in bold. PAM-site is shown in red in each wild type (WT) fragment. (PPTX 69 kb)

List of primers used for PCR amplification. (DOCX 17 kb)
