# Supplementary material for: Efficient targeted multiallelic mutagenesis in tetraploid potato (Solanum tuberosum) by transient CRISPR-Cas9 expression in protoplasts
Source: Plant Cell Rep. 2016 Oct 3;36(1):117–28. doi: 10.1007/s00299-016-2062-3 (PMC5206254; doi:10.1007/s00299-016-2062-3)
Supplement: Supplementary file 1 — Supplementary Allelic variation GBSS. Alignment of the four GBSS alleles of Kuras, determined by Sanger sequencing and compared to a publically available GBSS sequence (Gen Bank, accession no. A23741.1). (a) exon 8. (b) part of exon 9. Variations are marked with a red box. (PPTX 69 kb) [file 299_2016_2062_MOESM1_ESM.pptx]

## Slide 1
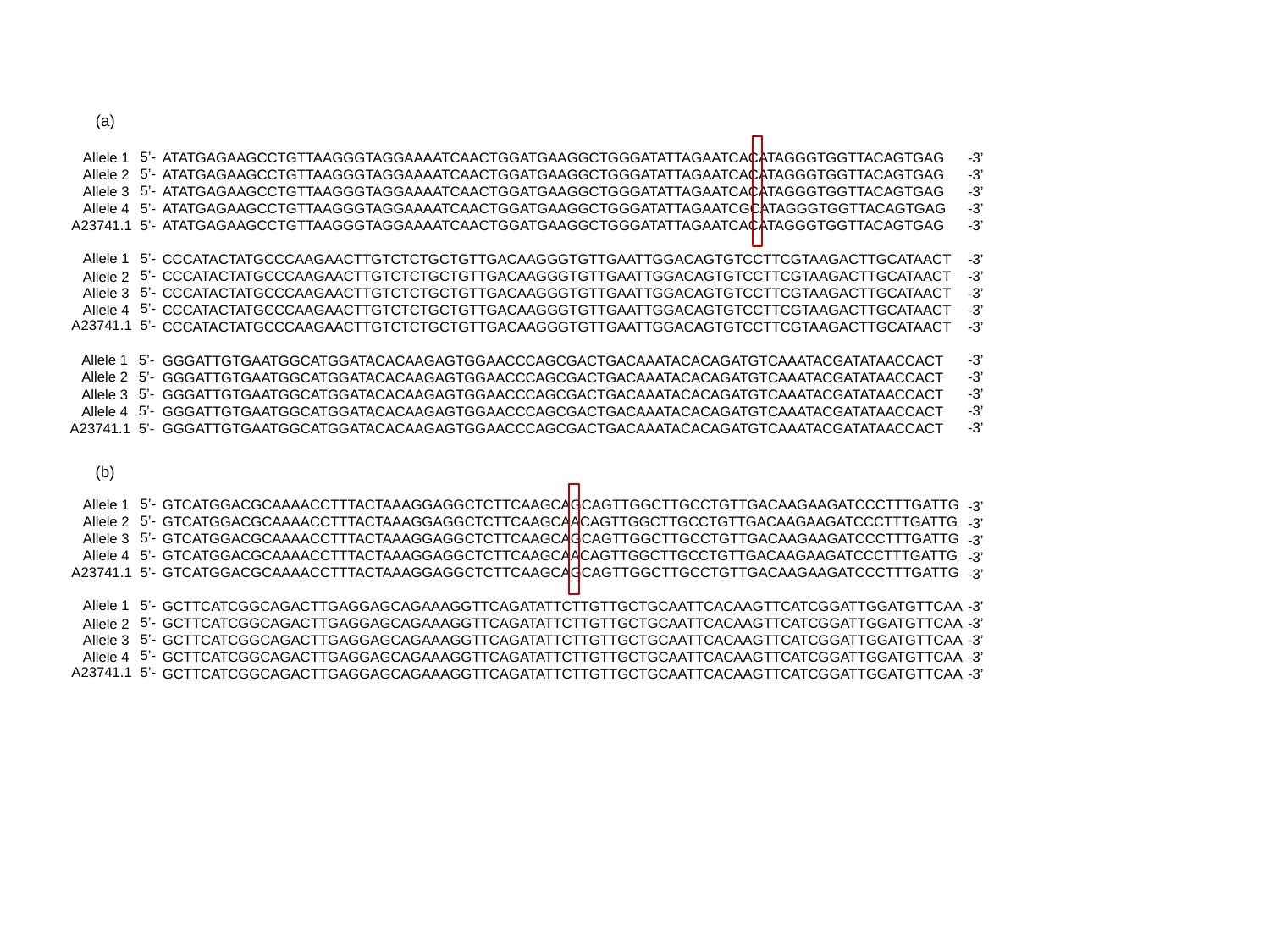

(a)
5’-
Allele 1
-3’
ATATGAGAAGCCTGTTAAGGGTAGGAAAATCAACTGGATGAAGGCTGGGATATTAGAATCACATAGGGTGGTTACAGTGAG
ATATGAGAAGCCTGTTAAGGGTAGGAAAATCAACTGGATGAAGGCTGGGATATTAGAATCACATAGGGTGGTTACAGTGAG
ATATGAGAAGCCTGTTAAGGGTAGGAAAATCAACTGGATGAAGGCTGGGATATTAGAATCACATAGGGTGGTTACAGTGAG
ATATGAGAAGCCTGTTAAGGGTAGGAAAATCAACTGGATGAAGGCTGGGATATTAGAATCGCATAGGGTGGTTACAGTGAG
ATATGAGAAGCCTGTTAAGGGTAGGAAAATCAACTGGATGAAGGCTGGGATATTAGAATCACATAGGGTGGTTACAGTGAG
CCCATACTATGCCCAAGAACTTGTCTCTGCTGTTGACAAGGGTGTTGAATTGGACAGTGTCCTTCGTAAGACTTGCATAACT
CCCATACTATGCCCAAGAACTTGTCTCTGCTGTTGACAAGGGTGTTGAATTGGACAGTGTCCTTCGTAAGACTTGCATAACT
CCCATACTATGCCCAAGAACTTGTCTCTGCTGTTGACAAGGGTGTTGAATTGGACAGTGTCCTTCGTAAGACTTGCATAACT
CCCATACTATGCCCAAGAACTTGTCTCTGCTGTTGACAAGGGTGTTGAATTGGACAGTGTCCTTCGTAAGACTTGCATAACT
CCCATACTATGCCCAAGAACTTGTCTCTGCTGTTGACAAGGGTGTTGAATTGGACAGTGTCCTTCGTAAGACTTGCATAACT
GGGATTGTGAATGGCATGGATACACAAGAGTGGAACCCAGCGACTGACAAATACACAGATGTCAAATACGATATAACCACT
GGGATTGTGAATGGCATGGATACACAAGAGTGGAACCCAGCGACTGACAAATACACAGATGTCAAATACGATATAACCACT
GGGATTGTGAATGGCATGGATACACAAGAGTGGAACCCAGCGACTGACAAATACACAGATGTCAAATACGATATAACCACT
GGGATTGTGAATGGCATGGATACACAAGAGTGGAACCCAGCGACTGACAAATACACAGATGTCAAATACGATATAACCACT
GGGATTGTGAATGGCATGGATACACAAGAGTGGAACCCAGCGACTGACAAATACACAGATGTCAAATACGATATAACCACT
5’-
Allele 2
-3’
5’-
Allele 3
-3’
5’-
Allele 4
-3’
-3’
A23741.1
5’-
Allele 1
5’-
-3’
5’-
-3’
Allele 2
5’-
-3’
Allele 3
5’-
Allele 4
-3’
A23741.1
5’-
-3’
5’-
-3’
Allele 1
-3’
5’-
Allele 2
-3’
5’-
Allele 3
-3’
5’-
Allele 4
-3’
A23741.1
5’-
(b)
5’-
Allele 1
GTCATGGACGCAAAACCTTTACTAAAGGAGGCTCTTCAAGCAGCAGTTGGCTTGCCTGTTGACAAGAAGATCCCTTTGATTG
GTCATGGACGCAAAACCTTTACTAAAGGAGGCTCTTCAAGCAACAGTTGGCTTGCCTGTTGACAAGAAGATCCCTTTGATTG
GTCATGGACGCAAAACCTTTACTAAAGGAGGCTCTTCAAGCAGCAGTTGGCTTGCCTGTTGACAAGAAGATCCCTTTGATTG
GTCATGGACGCAAAACCTTTACTAAAGGAGGCTCTTCAAGCAACAGTTGGCTTGCCTGTTGACAAGAAGATCCCTTTGATTG
GTCATGGACGCAAAACCTTTACTAAAGGAGGCTCTTCAAGCAGCAGTTGGCTTGCCTGTTGACAAGAAGATCCCTTTGATTG
GCTTCATCGGCAGACTTGAGGAGCAGAAAGGTTCAGATATTCTTGTTGCTGCAATTCACAAGTTCATCGGATTGGATGTTCAA
GCTTCATCGGCAGACTTGAGGAGCAGAAAGGTTCAGATATTCTTGTTGCTGCAATTCACAAGTTCATCGGATTGGATGTTCAA
GCTTCATCGGCAGACTTGAGGAGCAGAAAGGTTCAGATATTCTTGTTGCTGCAATTCACAAGTTCATCGGATTGGATGTTCAA
GCTTCATCGGCAGACTTGAGGAGCAGAAAGGTTCAGATATTCTTGTTGCTGCAATTCACAAGTTCATCGGATTGGATGTTCAA
GCTTCATCGGCAGACTTGAGGAGCAGAAAGGTTCAGATATTCTTGTTGCTGCAATTCACAAGTTCATCGGATTGGATGTTCAA
-3’
5’-
Allele 2
-3’
5’-
Allele 3
-3’
5’-
Allele 4
-3’
A23741.1
5’-
-3’
Allele 1
5’-
-3’
5’-
-3’
Allele 2
5’-
-3’
Allele 3
5’-
Allele 4
-3’
A23741.1
5’-
-3’
