# Supplementary figures and images for: Efficient targeted multiallelic mutagenesis in tetraploid potato (Solanum tuberosum) by transient CRISPR-Cas9 expression in protoplasts
Source: Plant Cell Rep. 2016 Oct 3;36(1):117–28. doi: 10.1007/s00299-016-2062-3 (PMC5206254; doi:10.1007/s00299-016-2062-3)

## Slide 1
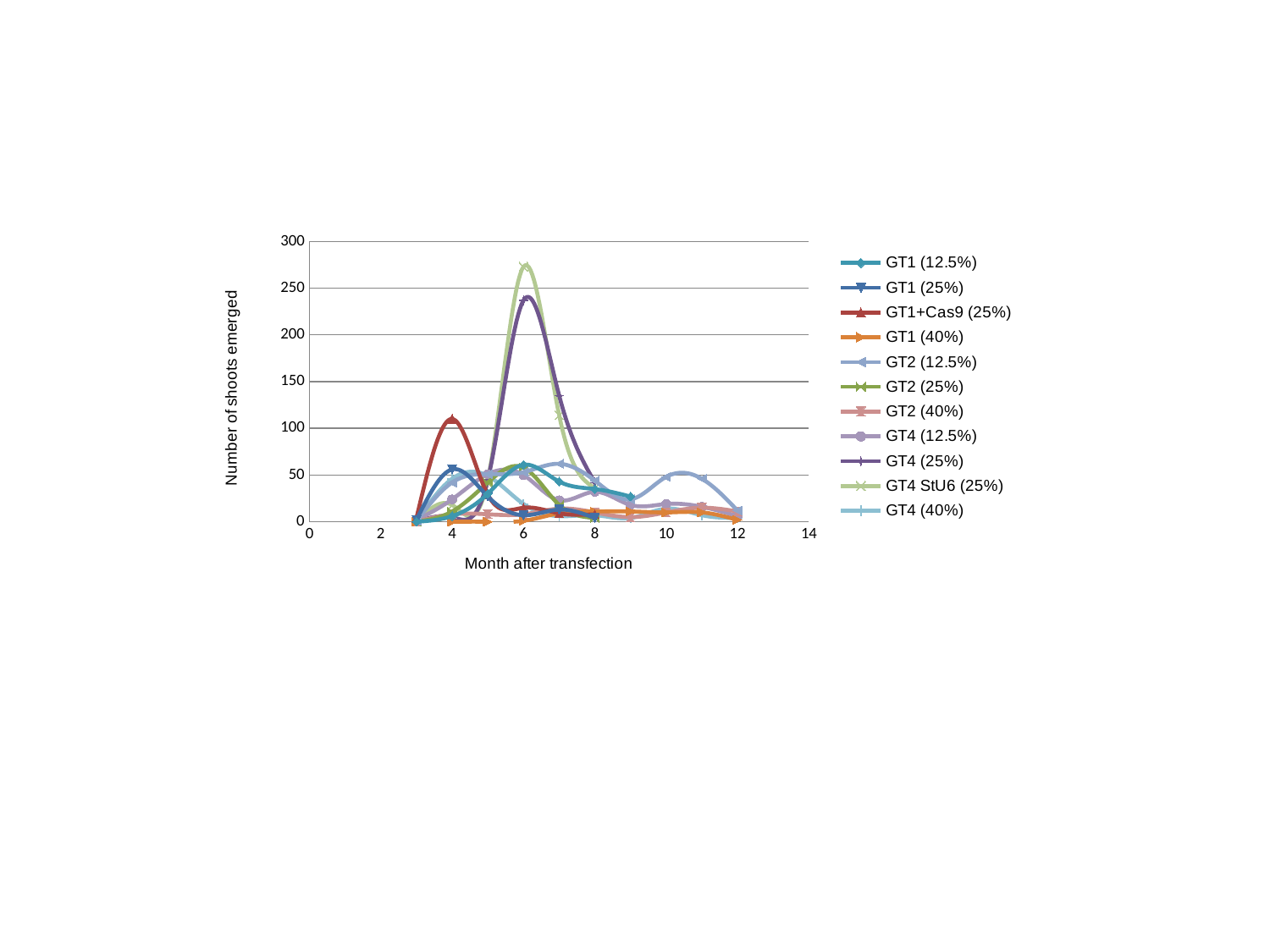

### Chart
| Category | GT1 (12.5%) | GT1 (25%) | GT1+Cas9 (25%) | GT1 (40%) | GT2 (12.5%) | GT2 (25%) | GT2 (40%) | GT4 (12.5%) | GT4 (25%) | GT4 StU6 (25%) | GT4 (40%) |
|---|---|---|---|---|---|---|---|---|---|---|---|

Supplement: Supplementary file 2 — Number of shoots regenerated per month after transfection. Guide sequences corresponding to target regions GT1, GT2 and GT4 at different transfection conditions. Shown within the parentheses’ is the PEG concentration used in the experiment. A total of 160,000 protoplasts were transfected in the 12.5 and 25 % PEG experimental setups while 100,000 protoplasts were transfected using the 40 % PEG experimental setup. (PPTX 167 kb) [file 299_2016_2062_MOESM2_ESM.pptx]
