# Supplementary material for: Efficient targeted multiallelic mutagenesis in tetraploid potato (Solanum tuberosum) by transient CRISPR-Cas9 expression in protoplasts
Source: Plant Cell Rep. 2016 Oct 3;36(1):117–28. doi: 10.1007/s00299-016-2062-3 (PMC5206254; doi:10.1007/s00299-016-2062-3)
Supplement: Supplementary file 3 — Genotyping of individual alleles. Lines with induced mutations in GT1, GT2 and GT4 target regions are presented. Deleted nucleotides are shown with hyphens and inserted nucleotides are shown in bold. PAM-site is shown in red in each wild type (WT) fragment. (PPTX 69 kb) [file 299_2016_2062_MOESM3_ESM.pptx]

## Slide 1
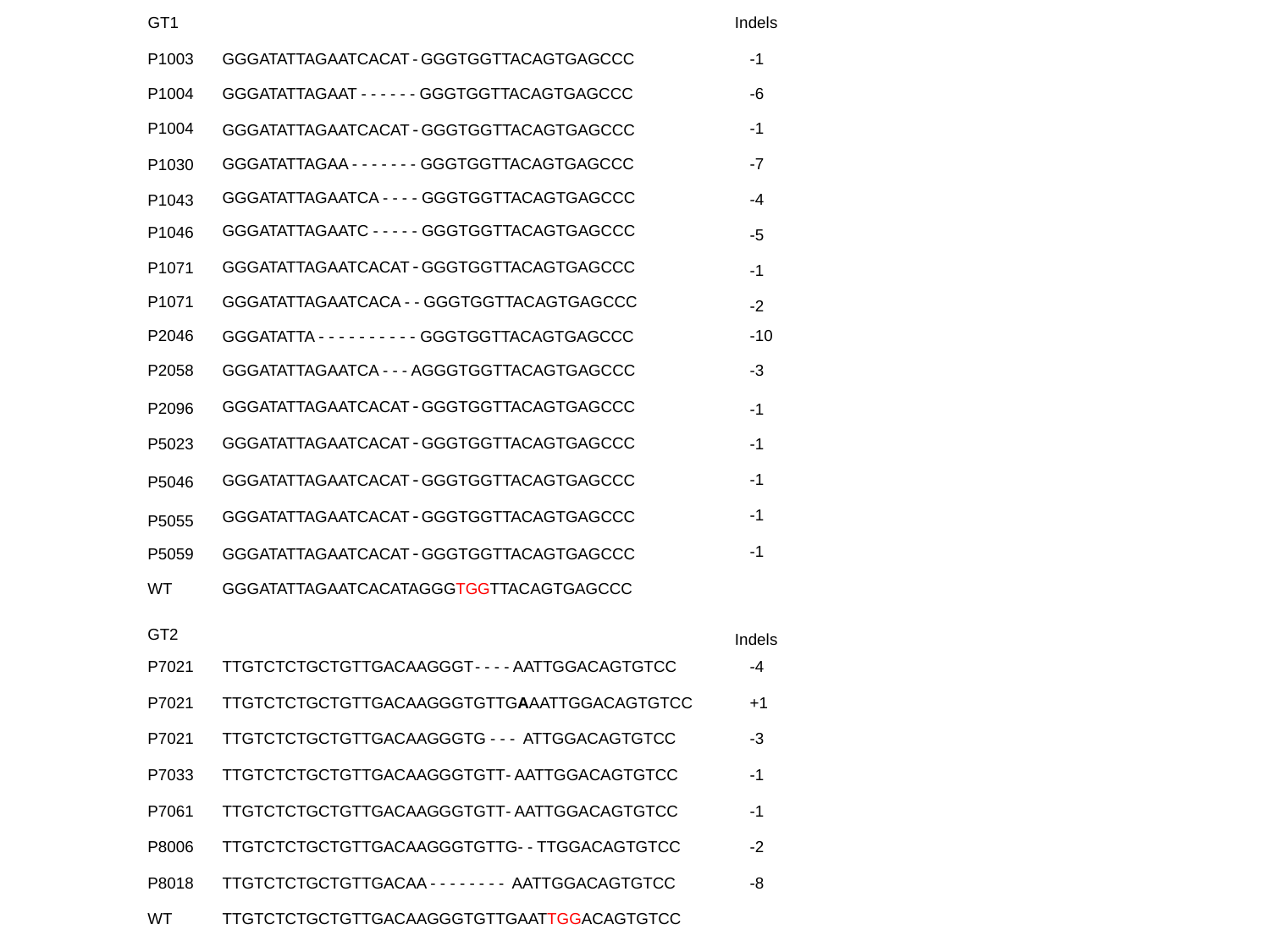

GT1
Indels
P1003
GGGATATTAGAATCACAT - GGGTGGTTACAGTGAGCCC
-1
P1004
GGGATATTAGAAT - - - - - - GGGTGGTTACAGTGAGCCC
-6
P1004
GGGATATTAGAATCACAT - GGGTGGTTACAGTGAGCCC
-1
GGGATATTAGAA - - - - - - - GGGTGGTTACAGTGAGCCC
-7
P1030
GGGATATTAGAATCA - - - - GGGTGGTTACAGTGAGCCC
-4
P1043
GGGATATTAGAATC - - - - - GGGTGGTTACAGTGAGCCC
P1046
-5
GGGATATTAGAATCACAT - GGGTGGTTACAGTGAGCCC
P1071
-1
P1071
GGGATATTAGAATCACA - - GGGTGGTTACAGTGAGCCC
-2
P2046
GGGATATTA - - - - - - - - - - GGGTGGTTACAGTGAGCCC
-10
P2058
GGGATATTAGAATCA - - - AGGGTGGTTACAGTGAGCCC
-3
GGGATATTAGAATCACAT - GGGTGGTTACAGTGAGCCC
P2096
-1
GGGATATTAGAATCACAT - GGGTGGTTACAGTGAGCCC
P5023
-1
GGGATATTAGAATCACAT - GGGTGGTTACAGTGAGCCC
-1
P5046
GGGATATTAGAATCACAT - GGGTGGTTACAGTGAGCCC
-1
P5055
GGGATATTAGAATCACAT - GGGTGGTTACAGTGAGCCC
-1
P5059
WT
GGGATATTAGAATCACATAGGGTGGTTACAGTGAGCCC
GT2
Indels
P7021
TTGTCTCTGCTGTTGACAAGGGT - - - - AATTGGACAGTGTCC
-4
P7021
TTGTCTCTGCTGTTGACAAGGGTGTTGAAATTGGACAGTGTCC
+1
P7021
TTGTCTCTGCTGTTGACAAGGGTG - - - ATTGGACAGTGTCC
-3
P7033
TTGTCTCTGCTGTTGACAAGGGTGTT - AATTGGACAGTGTCC
-1
P7061
TTGTCTCTGCTGTTGACAAGGGTGTT - AATTGGACAGTGTCC
-1
P8006
TTGTCTCTGCTGTTGACAAGGGTGTTG - - TTGGACAGTGTCC
-2
P8018
TTGTCTCTGCTGTTGACAA - - - - - - - - AATTGGACAGTGTCC
-8
WT
TTGTCTCTGCTGTTGACAAGGGTGTTGAATTGGACAGTGTCC

## Slide 2
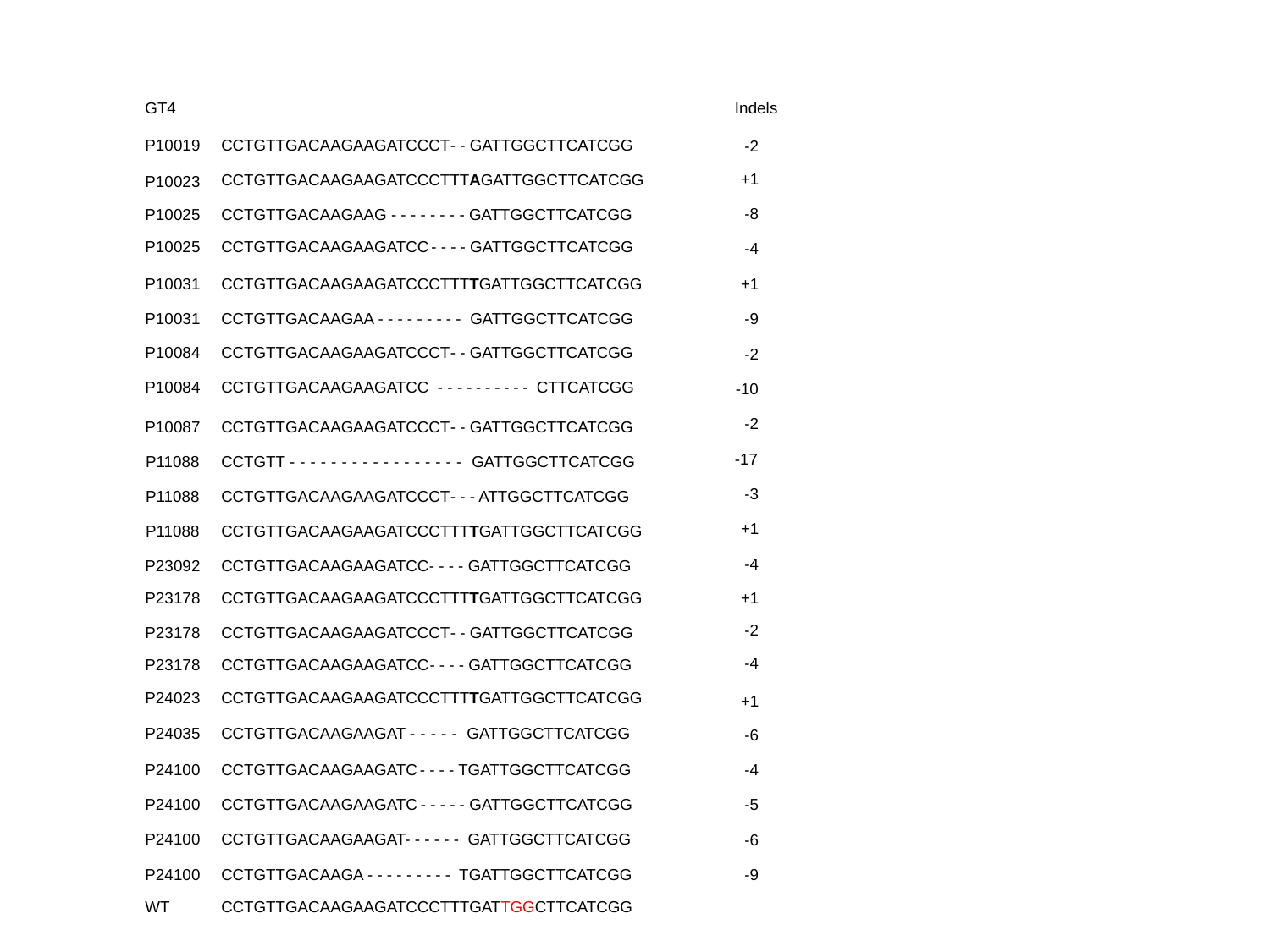

GT4
Indels
P10019
CCTGTTGACAAGAAGATCCCT - - GATTGGCTTCATCGG
-2
+1
CCTGTTGACAAGAAGATCCCTTTAGATTGGCTTCATCGG
P10023
-8
CCTGTTGACAAGAAG - - - - - - - - GATTGGCTTCATCGG
P10025
P10025
CCTGTTGACAAGAAGATCC - - - - GATTGGCTTCATCGG
-4
P10031
CCTGTTGACAAGAAGATCCCTTTTGATTGGCTTCATCGG
+1
P10031
CCTGTTGACAAGAA - - - - - - - - - GATTGGCTTCATCGG
-9
P10084
CCTGTTGACAAGAAGATCCCT - - GATTGGCTTCATCGG
-2
P10084
CCTGTTGACAAGAAGATCC - - - - - - - - - - CTTCATCGG
-10
-2
P10087
CCTGTTGACAAGAAGATCCCT - - GATTGGCTTCATCGG
-17
P11088
CCTGTT - - - - - - - - - - - - - - - - - GATTGGCTTCATCGG
-3
P11088
CCTGTTGACAAGAAGATCCCT - - - ATTGGCTTCATCGG
+1
P11088
CCTGTTGACAAGAAGATCCCTTTTGATTGGCTTCATCGG
-4
P23092
CCTGTTGACAAGAAGATCC - - - - GATTGGCTTCATCGG
P23178
CCTGTTGACAAGAAGATCCCTTTTGATTGGCTTCATCGG
+1
-2
P23178
CCTGTTGACAAGAAGATCCCT - - GATTGGCTTCATCGG
-4
P23178
CCTGTTGACAAGAAGATCC - - - - GATTGGCTTCATCGG
P24023
CCTGTTGACAAGAAGATCCCTTTTGATTGGCTTCATCGG
+1
P24035
CCTGTTGACAAGAAGAT - - - - - GATTGGCTTCATCGG
-6
-4
P24100
CCTGTTGACAAGAAGATC - - - - TGATTGGCTTCATCGG
P24100
CCTGTTGACAAGAAGATC - - - - - GATTGGCTTCATCGG
-5
P24100
CCTGTTGACAAGAAGAT- - - - - - GATTGGCTTCATCGG
-6
P24100
CCTGTTGACAAGA - - - - - - - - - TGATTGGCTTCATCGG
-9
WT
CCTGTTGACAAGAAGATCCCTTTGATTGGCTTCATCGG
