# Supplementary material for: Efficient targeted multiallelic mutagenesis in tetraploid potato (Solanum tuberosum) by transient CRISPR-Cas9 expression in protoplasts
Source: Plant Cell Rep. 2016 Oct 3;36(1):117–28. doi: 10.1007/s00299-016-2062-3 (PMC5206254; doi:10.1007/s00299-016-2062-3)
Supplement: Supplementary file 4 — List of primers used for PCR amplification. (DOCX 17 kb) [file 299_2016_2062_MOESM4_ESM.docx]

| **Primer name** | **Primer sequence** |
| --- | --- |
| StGBSSExf | 5’-CCTCTTCTCAATCTTCCTGATGAATTCAG-3´ |
| StGBSSExr | 5’-AGAGCCTCCTTTAGTAAAGGTTTTGCGTC-3´ |
| StGBSSExon3f | 5’-GCATCCATAACATTGCCTACC-3´ |
| StGBSSExon3r | 5’-TGAACATCCAATCCGATGAAC-3 |
| StGBSSexon1f-FAM, , | FAM-5’-ACTGGATGAAGGCTGGGATA 3’ |
| StGBSSexon1f-VIC | VIC-5’-ACTGGATGAAGGCTGGGATA 3’ |
| StGBSSexon1r | 5’-ATTTGTCAGTCGCTGGGTTC 3’ |
| StGBSS(GT4)f-FAM | FAM-5’-TCTCTATACAGGTCATGGACG-3’ |
| StGBSS(GT4)f-VIC | VIC-5’-TCTCTATACAGGTCATGGACG-3’ |
| StGBSS(GT4)r | 5’- GCAGCAACAAGAATATCTGAAC-3’ |
| StGBSSexon1f | 5’-ACTGGATGAAGGCTGGGATA 3’ |
| StGBSS(GT4)f | 5’-TCTCTATACAGGTCATGGACG-3’ |
